# Exploring Alzheimer's Disease Subtype Replicability

**DOI:** 10.1002/alz70855_103465

**Published:** 2025-12-23

**Authors:** Alexei Taylor, Alexandra Sahl, Hansoo Chang, Ana Ferariu, Lei Wang, Wenxin Jiang, Corey T. McMillan, Michelle D. Shardell, Fengqing (Zoe) Zhang

**Affiliations:** ^1^ Drexel University, Philadelphia, PA, USA; ^2^ Ohio State University, Columbus, OH, USA; ^3^ Northwestern University, Evanston, IL, USA; ^4^ University of Pennsylvania, Philadelphia, PA, USA; ^5^ University of Maryland, Baltimore, MD, USA

## Abstract

**Background:**

Alzheimer's disease (AD) cognitive decline is correlated with tau neuropathology, which can follow a typical spatiotemporal pattern spreading from the medial temporal lobe to the lateral temporal, parietal, frontal, and occipital lobes, at varying levels of severity. However, atypical patterns have also been observed in postmortem studies. Recently, more studies have used hypothesis‐driven partitioning informed by postmortem findings or data‐driven clustering with in‐vivo imaging data to identify AD subtypes with distinct clinical characteristics. However, major challenges remain: (i) AD subtypes are poorly defined, and are difficult to compare, especially at the individual‐level, (ii) Distinct pathological tau and gray matter atrophy patterns characterize AD subtypes, but the unique spatiotemporal relationship is unknown. Thus, the present study proposes a framework to aid interpretation of subtype replicability by utilizing hypothesis‐driven postmortem data to guide data‐driven clustering.

**Method:**

Data from the Alzheimer's Disease Neuroimaging Initiative (ADNI) was used. The likelihood of an AD subtype was computed for each participant by counting their subtype assignment across common hypothesis‐driven protocols. Semi‐supervised clustering made partial use of this likelihood to cluster the ADNI data, and assignment consistency and stability were compared to unsupervised clustering using a nested cross‐validation design. Clustering from samples with cross‐sectional tau features (*n* =  222), and separately, atrophy‐related features (*n* =  527) were then evaluated.

**Result:**

When evaluated on a held‐out test set of participants with a high likelihood for different AD subtypes, semi‐supervised clustering trained on a portion of “high‐likelihood participants” demonstrated consistent subtype prediction on the held‐out test set (median adjusted rand index of 0.57 when using tau features, and 0.12 when using atrophy‐related features), whereas typical unsupervised methods (e.g., hierarchical, k‐means) produced chance‐level clustering.

**Conclusion:**

The results demonstrate the risk of relying on unsupervised clustering for interpreting AD subtypes and recommend a framework for defining the subtypes, training clustering, and evaluating performance based on hypothesis‐driven definitions grounded in the original postmortem findings.

